# Improving rule-based classification using Harmony Search

**DOI:** 10.7717/peerj-cs.188

**Published:** 2019-11-18

**Authors:** Hesam Hasanpour, Ramak Ghavamizadeh Meibodi, Keivan Navi

**Affiliations:** Department of Computer Science and Engineering, Shahid Beheshti University, Tehran, Iran

**Keywords:** Apriori algorithm, CBA algorithm, Harmony Search

## Abstract

Classification and associative rule mining are two substantial areas in data mining. Some scientists attempt to integrate these two field called rule-based classifiers. Rule-based classifiers can play a very important role in applications such as fraud detection, medical diagnosis, etc. Numerous previous studies have shown that this type of classifier achieves a higher classification accuracy than traditional classification algorithms. However, they still suffer from a fundamental limitation. Many rule-based classifiers used various greedy techniques to prune the redundant rules that lead to missing some important rules. Another challenge that must be considered is related to the enormous set of mined rules that result in high processing overhead. The result of these approaches is that the final selected rules may not be the global best rules. These algorithms are not successful at exploiting search space effectively in order to select the best subset of candidate rules. We merged the Apriori algorithm, Harmony Search, and classification-based association rules (CBA) algorithm in order to build a rule-based classifier. We applied a modified version of the Apriori algorithm with multiple minimum support for extracting useful rules for each class in the dataset. Instead of using a large number of candidate rules, binary Harmony Search was utilized for selecting the best subset of rules that appropriate for building a classification model. We applied the proposed method on a seventeen benchmark dataset and compared its result with traditional association rule classification algorithms. The statistical results show that our proposed method outperformed other rule-based approaches.

## Introduction

The availability of a huge amount of raw data has created an immense opportunity for knowledge discovery and data mining research to play an essential role in a wide range of applications such as industry, financial forecasting, weather forecasting and healthcare.

Classification is one of the most important areas in data mining that has applied in many applications such as bioinformatics, fraud detection, loan risk prediction, medical diagnosis, weather prediction, customer segmentation, target marketing, text classification and engineering fault detection. Association Rule Mining (ARM) is another popular and substantial technique in machine learning and data mining that introduced by Agrawal, Imieliński & Swami (1993), and since that remained one of the most active research areas in machine learning and knowledge discovery. Association rule mining finds interesting relationships among large sets of data items. Association rules show attribute value conditions that occur frequently together in a given data set. Association rules provide information of this type in the form of if-then statements. Unlike the if-then rules of logic, association rules are intrinsically probabilistic and are computed from the data. The ARM is a powerful exploratory technique with a wide range of applications including marketing policies, medical domain (Ilayaraja & Meyyappan, 2013; Shin et al., 2010), financial forecast, credit fraud detection (Sarno et al., 2015) and many other areas. There are a number of famous association rule mining algorithms that are accessible to researchers (Agrawal, Imieliński & Swami, 1993; Burdick, Calimlim & Gehrke, 2001; Scheffer, 2001a).

There is some evidence that integration benefits of classification and association rule mining together can result in more accurate and efficient classification models than traditional classification algorithms (Ma & Liu, 1998). Producing concise and accurate classifier by utilizing association rule mining is one of the attractive domain for data mining and machine learning researchers.

A typical associative classification system is constructed in two stages:

1. discovering all the association rules inherent in a database.
2. Selecting a small set of relevant association rules to construct a classifier.

In the first step, some algorithms use Apriori algorithm for rule generation and some other algorithms use other approaches such as FOIL (First Order Inductive Learner). Mazid, Ali & Tickle (2009) compared the performance between the rule-based classification and association rule mining algorithm based on their classification performance and computational complexity. They concluded that Apriori is a better choice for rule-based mining task in terms of accuracy and computational complexity.

Usually a lot of rules are generated in the first step and the main issue on second step is that how to efficiently find out a small number of high-quality rules and how to generate a more accurate classifier. It must be noted that some researchers focus on the first step and try to find a minimal class association rule set (Li, Shen & Topor, 2002), but our focus is on the second step.

Traditional algorithms use greedy approaches for selecting a small subset of generated rules for building a classifier. By using this approach, the selected rules are not the best subset of possible rules. Another challenge is that the resulted rules bias to prevalent classes and classification the rare instances is a major problem. consequently, test samples belonging to the small classes are misclassified as prevalent classes (Chen, Hsu & Hsu, 2012; Sun, Wong & Kamel, 2009). Sometimes rules with low support and very high confidence are effective in identifying rare events.

In this paper, we present an association rule-based classification method to obtain an accurate and compact rule-based classifier. We used the Apriori algorithm for rule generation and Harmony Search for selecting the best subset of rules that can build a classifier.

The plan of this paper is as follows: at first, we present the necessary information related to rule-based classification. In the next section, we describe the proposed method. The Results section shows the induced results and, finally, the Discussion section concludes the study.

## Preliminaries

### Apriori algorithm and interesting measures

Apriori is a standard and well-known basic algorithm in association rule mining that is used for mining frequent itemsets in a set of transactions. It was first introduced by Agrawal and Srikant (Agrawal, Imieliński & Swami, 1993). The APRIORI-C is another Apriori-based algorithm that drives rules according to the parameters minimal confidence and minimal support of a rule (Jovanoski & Lavrač, 2001). Predictive Apriori (Scheffer, 2001b) in another algorithm motivated by Apriori and unlike the confidence related focus of Apriori tries to maximizes the expected accuracy of an association rule on unseen data. While Apriori sorts the rules based on confidence only, Predictive Apriori considers both the confidence and support in ranking the rules.

Nahar et al. considered three rule generation algorithms—Apriori, Predictive Apriori and Tertius- for extraction the meaningful factors for particular types of cancer (Nahar et al., 2011) and heart disease (Nahar et al., 2013). Their experimental results showed that Apriori is the most beneficial association rule mining algorithm.

Apriori algorithm can produce a lot of rules, but much of them are superfluous. To select appropriate rules from the set of all possible rules, constraints on various measures of interestingness can be used. Support and confidence are two measures of rule interestingness that mirror the usefulness and certainty of a rule respectively (Agrawal et al., 1996). The support is the percentage of the total number of records of transactions that include all items in the antecedent (if) and consequent (then) parts of the rule. Frequent itemsets are those itemsets that their frequency is greater than a predefined minimum support (Minsup). Confidence is the ratio of the number of transactions that include all items in the consequent, as well as the antecedent (the support) to the number of transactions that include all items in the antecedent. In other words, confidence is the accuracy of the rule and usually is used in Apriori for ranking the rules. The task of association rule mining is to generate all association rules from the set of transactions that have a support greater than Minsup and confidence greater than Mincon. Since we need to discover the relationship between input attributes and class label, we need to find all the rules of the form A → B that antecedent part of the rule includes of some item and the consequent part can just be the class items.

High support and high confidence rules are not necessarily interesting. Instead of using only support and confidence, we also used lift measure as a metric for evaluating the significance and reliability of association rules. Lift is the ratio of Confidence to Expected Confidence. Hence, Lift is a value that gives us information about the increase in the probability of the consequent given antecedent part of a rule. A lift ratio larger than 1.0 implies that the relationship between the antecedent and the consequent is more significant than would be expected and make those rules potentially useful for predicting the consequent in unseen instances. The larger the lift ratio, the more significant the association.

(1) \begin{eqnarray*}& & Support \left( X\Longrightarrow Y \right) =P \left( X\cap Y \right) \end{eqnarray*}

(2) \begin{eqnarray*}& & Confidence \left( X\Longrightarrow Y \right) =P \left( Y{|}X \right) = \frac{Support(X\cap Y)}{Support(X)} \end{eqnarray*}

(3) \begin{eqnarray*}& & Lift \left( X,Y \right) = \frac{P(X\cap Y)}{P \left( x \right) \mathrm{ \ast }P(Y)} .\end{eqnarray*}

Another issue that must be considered is related to the type of dataset that is appropriate for applying the Apriori algorithm. Consider a dataset for supervised learning which contains observations of a class label variable and a number of predictor variables. Such a dataset can be converted into an appropriate format for association rule mining if both the class label and the predictors are of the categorical type. Since our benchmark datasets contain continuous variables, we must use a method for handling numeric attributes. There are some methods for this purpose. A traditional method is discretization that can be static or based on the distribution of data. We used a method proposed by Tsai, Lee & Yang (2008).

### Associative rules for classification

In recent years, some researchers tried to combine association rule mining and classification (Cano, Zafra & Ventura, 2013; Li, Han & Pei, 2001; Ma & Liu, 1998; Wang, Zhou & He, 2000; Wang & Wong, 2003; Yin & Han, 2003). Their experiments show that this approach achieves better accuracy than conventional classification algorithms such as C4.5. The reason is that the associative classifier is composed of high-quality rules, which are generated from highly confident event associations that reflect the close dependencies among events.

The Classification Based on Association rules (CBA) algorithm is one of the first efforts for combining of classification and association rule mining (Ma & Liu, 1998). This algorithm will describe with details in the next section. Li, Han & Pei (2001) suggested a weighted *χ*2 analysis to perform a Classification based on Multiple Association Rules (CMAR). Unlike the CBA algorithm, the CMAR algorithm uses all the rules that cover the example to be classified instead of using just one rule.

Yin & Han (2003) propose the CPAR (Classification based on Predictive Association Rules) rule-based classification algorithm CPAR doesn’t generate a large number of candidate rules as in conventional associative classification. It pursues a greedy algorithm to produce rules directly from training data and uses the best K rules in prediction time.

An advantage of associative classifiers is that they are rule-based and thus lend themselves to be more easily understood by humans. As previously stated, a classification system is built in two phase. In the first stage, the learning target is to discover the association patterns inherent in a database (also referred to as knowledge discovery). In the second stage, the goal is to select a small set of relevant association patterns to construct a classifier given the predictor attributes. To produce the best classifier out of the entire set of rules, we need to consider all the feasible subsets of rules and selecting the most accurate subset. This is clearly impractical.

In the classification phase, some methods such as (Ma & Liu, 1998; Thabtah, Cowling & Peng, 2004; Wang, Zhou & He, 2000), simply select a rule with a maximal user-defined measure, such as confidence. If there is no rule covering the example, then usually the prevalent class is taken to be the predicted class. However, identifying the most effective rule at classifying a new case is a big challenge. When classifying a new data object, it may have more rules that satisfy the test conditions and using them may increase the prediction accurately (Li, Han & Pei, 2001).

### CBA algorithm

Classification Based on Associations (CBA) algorithm is one of the first algorithms to bring up the idea of classification using association rules (Ma & Liu, 1998). CBA implements the famous Apriori algorithm (Agrawal, Imieliński & Swami, 1993) in order to discover frequent items. Once the discovery of frequent items finished, CBA proceeds by converting any frequent item that passes the Minconf into a rule in the classifier. At the rule generation phase, CBA selects a special subset of association rules whose right-hand-side are restricted to the classification class attribute. This subset of rules is called class association rules (CARs). At the next step, the CBA algorithm builds a classifier using CARs. At this step, CBA uses a heuristic approach and sorts the rules according to their confidence and selects top rules that cover the training samples.

The algorithm first selects the best rule (rule having the highest confidence), then eliminates all the covered examples. If at least one example satisfied the rule conditions, then that rule is appended to the final rules. This procedure is repeated until there are no more rules to select or there are no more examples to cover. The algorithm then stops and returns the classifier in the form of an IF-THEN-ELSE rule list. One challenge with this approach is that selecting the best rules may be not the best subset of rules.

**Figure 1:**
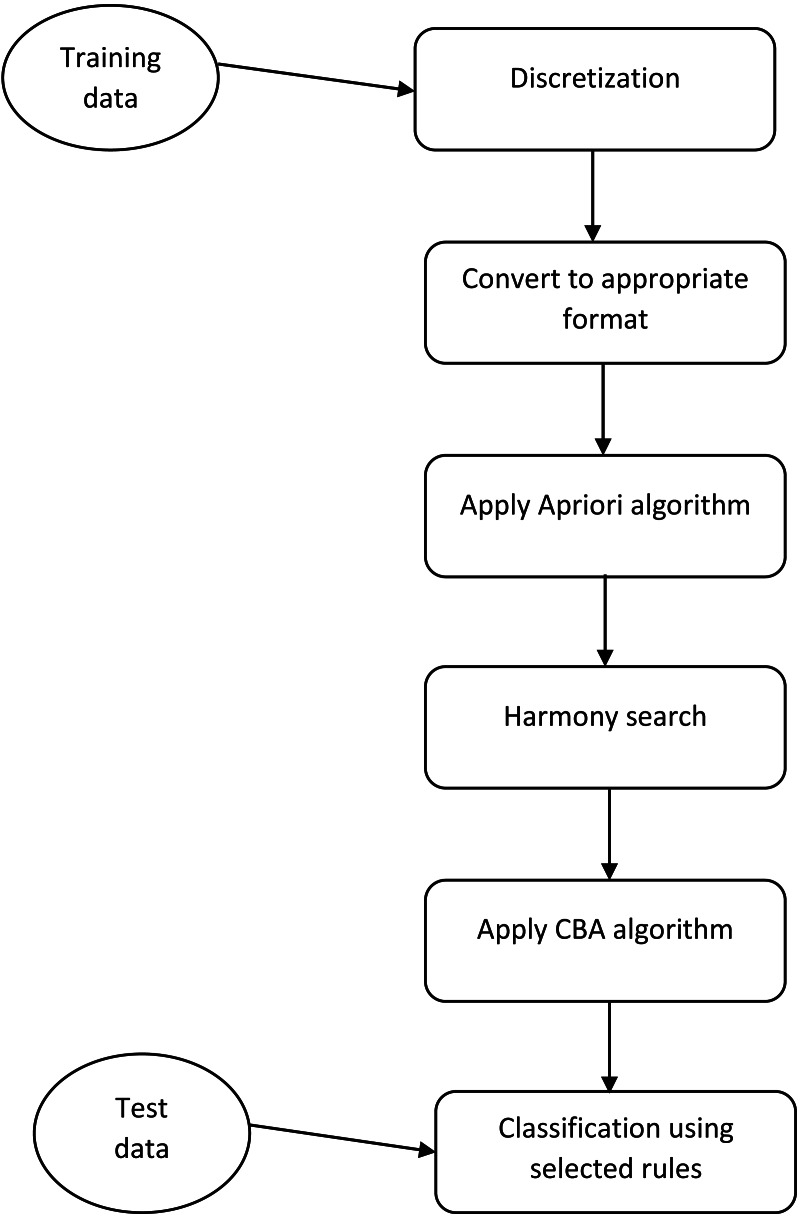
The framework of the proposed method. This figure shows what steps must be done for implementation of the proposed method.

The CBA system follows the original association rule model and uses a single Minsup in its rule generation. It seems that this is inadequate for mining of CARs because class frequency distributions in many practical classification datasets is unbalanced. We used the CBA algorithm with three little changes. The first change is that we use multiple Minsup than can be useful for imbalanced datasets. The second change is that in the original CBA algorithm once each sample is covered by a rule, it is removed from the samples; we defined a parameter called Delta. This parameter defined that how many times each sample must be covered to remove from the samples (Li, Han & Pei, 2001). This approach leads to the generation of more rules. The third change occurs in the classification phase. In the classification phase of the original CBA algorithm, the rule with maximum confidence that covers the test conditions defines the class label of a test sample. We select the top K (a predefined parameter) rules from each class that covers the test sample conditions and determined the class label according to the sum of the confidence of selected rules.

All data preprocessing and analyse were conducted using Matlab version 2014a (The MathWorks Inc., Natick, MA, USA).

### Proposed method

The proposed method of rule selection based on HS are depicted in Fig. 1. At the initial step, we did some preprocessing on each dataset. One of the main preprocessing is discretization of continuous features. We applied a discretization algorithm based on a class-attribute contingency coefficient that was proposed by Tsai, Lee & Yang, (2008). After discretization, we convert each dataset to the appropriate format such that the value of each feature can be True (1) or False (0). For this aim, if a feature is converted to N different discrete values, we produce N feature. After the conditions are satisfied for the Apriori algorithm, we run this algorithm for each class with different Minsup and Minconf. The main novelty of our study is in the next step. As previously was mentioned, the Apriori algorithm produces many rules and CBA algorithm uses a greedy algorithm for selecting a subset of produced rules for building a classifier. Using greedy approaches cause the selected rules to not be the best subset of rules.

We believe that population-based evolutionary algorithms fit well to the rule selection problem. Harmony Search (HS) is a population-based stochastic search algorithm that inspired by the musical process of searching for a perfect state of harmony (Geem, Kim & Loganathan, 2001). The harmony in music is analogous to the optimization solution vector, and the musician’s improvisations are similar to local and global search methods in optimization techniques When a musician is improvising, he has three choices: (1) to execute any pitch from memory; (2) to execute a pitch next to any other in his memory; (3) to execute a random pitch from the range of all possible pitches. These three options are employed in the HS algorithm by means of three main parameters: Harmony Memory (HM), Harmony Memory Consideration Rate (HMCR), and Pitch Adjustment Rate (PAR). The HMCR is defined as the probability of selecting a component from the present HM members. The PAR determines the probability of a candidate from the HM to be mutated. The steps in the procedure of HS are as follows:

Step 1. Initialize a harmony memory (HM). The initial HM consists of a given number of randomly generated solutions to the optimization problems under consideration.

Step 2. Improvise a new harmony from HM.

Step 3. Update the HM. If the new harmony is better than the worst harmony in HM, then include the new harmony in the HM and exclude the worst harmony from the HM.

Step 4. If the stopping criteria we not satisfied, go to step 2.

HS has been successfully applied to various discrete optimization problems such as Maximum Clique Problem (Afkhami, Ma & Soleimani, 2013), traveling salesperson problem (Geem, Kim & Loganathan, 2001), tour routing (Geem, Tseng & Park, 2005), water network design (Geem, 2006), dynamic relocation of mobile base stations in wireless sensor networks (Moh’d Alia, 2017), and others.

In binary HS, the size of each solution equals the number of candidate’s rules. For example, if the Apriori algorithm produces 100 rules that satisfy Minsup and Minconf conditions then the size of each solution in HS will be equal to 100. Each solution consists of a binary vector of rule incidences, indicating exclusion (0) or inclusion (1) of the rule in the combination.

The standard Harmony Search (HS) is not suitable for binary representations. This is due to the pitch adjusting operator not being able to perform the local search in the binary space. Therefore we used the implementation of HS that proposed by Afkhami, Ma & Soleimani (2013).

We run the HS algorithm with the following parameters: maximum number of iterations = 20, harmony memory size = 100, Number of new harmonies = 20, harmony memory consideration rate = 0.75.

We used Harmony Search, a music-inspired stochastic search algorithm, for selecting the best subset of rules as a classifier. One of the important section in any meta-heuristic algorithm is the calculation of cost function. For this aim, we apply a modified version of the CBA algorithm on the selected rules and calculate the error rate of applying the resulted rules on the training and validation data. At final, the solution with the minimum cost value is selected and this solution (a subset of rules) applies on the test data. It is obvious that the proposed flowchart is shown for one fold of cross-validation. In K-fold cross-validation, this approach must be repeated for K times, until all the samples in the dataset are used for the test data. The pseudo code of the proposed method is shown in Table 1.

**Table 1:** Pseudo code of the proposed method. This pseudo code supposes that we have training input, training output, test input, validation input and validation output. The code shows that how we build a rule-based classifier and determine the test data output.


For *i* = 1 to K fold
Determine Traininput, Trainoutput, Testinput, Testoutput, Valinput and Valoutput
Finalrules={};
For *j* = 1 to number_class
Rulesj= apply Apriori algorithm(traininput, Minsupj,Minconj,class j )
Finalrules= append Rulesj to Finalrules
End %for j
Selected_rules=Apply harmony search algorithm (Finalrules, Traininput, Trtainoutput, Valinput, Valoutput)
Testoutput= apply selected_rules on Testinput
End %for i

Time complexity of the Apriori algorithm and association rule mining is a critical challenge that must be considered (Cano, Luna & Ventura, 2013; Cano, Zafra & Ventura, 2014; Luna et al., 2016; Thabtah, Cowling & Hammoud, 2006). As its time complexity is exponential, we can do some preprocessing activity to decrease the running time. First of all, we can apply feature selection before applying Apriori algorithm. Feature selection can be done before or after of discretization. The second thing that we can do is related to the size of the rules. As small rules are favorable, we can limit the size of items that appear in a rule and consequently decrease the running time of Apriori algorithm.

## Results

We applied the proposed method on seventeen benchmark dataset and compare its result with traditional association rule classification algorithms. We compared our proposed method with the CPAR, CBA and C4.5 algorithms that are famous in rule-based classification (Ma & Liu, 1998; Quinlan, 1993; Yin & Han, 2003) The characteristic of the used datasets are shown in Table 2. We selected datasets with a verity of size in samples, attributes and number of classes.

**Table 2:** Description of used datasets. Each row shows the specifications of a used dataset including the name of the dataset, the number of features, the number of classes and distribution of classes.

id	Dataset	# of Data items	# of features	# of class	distribution
1	Iris	150	4	3	50–50–50
2	Galaxy	323	4	7	51–28–46–38–80–45–35
3	Wine	178	13	3	59–71–48
4	Tictactoe	958	9	2	626–332
5	SAHeart	462	9	2	160–302
6	Car	1,728	6	4	1,210–384–65–69
7	Breast cancer	699	19	2	458–241
8	Yeast	1,484	8	10	244–429–463–44–35–51–163 –30–20–5
9	Balance scale	625	4	3	49–288–288
10	lymphography	148	18	4	2–81–61–4
11	Haberman	306	3	2	225–81
12	Mammographic	830	5	2	427–403
13	phoneme	5,404	5	2	3,818–1,586
14	Pima	768	8	2	267–500
15	German	1,000	20	2	700–300
16	Monks-2	432	6	2	142–290
17	Monks-3	432	6	2	228–204

To run the experiments, a stratified five-fold cross-validation was used to produce a reliable accuracy. Cross-validation is a standard evaluation measure for calculating error rate on data in machine learning. At each run, we split each dataset to five parts, three part for training, one part for validation and one part for testing. To increase reliability, the experiments for each dataset have been repeated 10 times and the average of results were reported.

The result of the proposed method is shown in Table 3. As the results show, at four dataset decision tree gain the best accuracy, CPAR algorithm have the highest accuracy at the five datasets and our proposed method is the best at the seven datasets out of nine datasets. In one dataset, all algorithms are perfect and gain equal accuracy. The CBA algorithm is not the best in none of the datasets and in all of the datasets our proposed method outperformed the CBA algorithm. It must be noted that the results of decision tree, CBA and CPAR algorithms are reproduced on the same partitions.

**Table 3:** Experiment results based on 20 repetitions of 10-fold cross validation. Each row of the table shows the accuracy of applying four rule-based classification algorithm on a dataset.

Id	dataset	Decision tree	CBA	CPAR	Proposed method
1	Iris	91.33	94.6	94.67	**96.67**
2	Galaxy	**68.73**	56.66	60.37	61.12
3	Wine	86.52	95.12	94.38	**97.19**
4	Tictactoe	91.22	80.48	**97.39**	92.81
5	SAHeart	64.94	66.45	71.21	**73.43**
6	Car	94.68	73.38	**95.78**	80.22
7	Breast cancer	92.85	85.69	95.42	**96.28**
8	Yeast	54.99	52.96	**57.61**	56.27
9	Balance scale	**77.44**	72.20	71.36	73.76
10	Lymphography	73.65	57.43	**83.11**	74.32
11	haberman	71	73	73.84	**75.16**
12	mammographic	81.08	80.81	81.49	**83.20**
13	phoneme	**85.79**	70.65	70.73	77.22
14	pima	71.08	71	64.84	**72.01**
15	German	**73.1**	67	71.40	68.7
16	Monks 2	73.61	60.49	**80.79**	64.58
17	Monk 3	1	1	1	1
Mean Rank		2.55	3.5	2.14	1.79

We used the Friedman test (Friedman, 1940) as an appropriate choice for comparing multiple classification algorithms (Brazdil & Soares, 2000; Demšar, 2006). The Friedman test is a non-parametric statistical test developed by Milton Friedman (Friedman, 1937; Friedman, 1940). Similar to the parametric repeated measures ANOVA, it is used to detect differences between groups when the dependent variable being measured is ordinal. It must be noted that classification algorithms are ranked on each of the datasets and then the Friedman test is applied.

The last row of Table 3 shows the mean rank for each of the algorithms. As the results show, proposed method gained the best position and CBA has the worst one. The results also shows that there is an overall statistically significant difference between the mean ranks of the related algorithms (*P* = 0.0005).

The reported accuracy of other studies may be different in some algorithms from our ones. One of the main reason for this conflict is that we had no information about the discretization algorithm, in particularly the number of ranges used to discretize continuous attributes. Using different discretization approaches can result in different outputs.

## Discussion

This research has focused on the application of computational intelligence in association rule mining-based classifiers. Although rule-based classification algorithms have high classification accuracy, but some of them suffer from a critical limitation. They used a heuristic approach for selection a subset of rules for building a classifier. It is obvious that the selected rules may not be the best subset of possible rules. Another challenge of existing algorithms is related to rare class. Using greedy approaches, the resulted rules bias to prevalent classes and classification the rare instances is a major problem.

We combined the Apriori, CBA and Harmony Search algorithms in order to build a rule-based classifier that has a high prediction accuracy. We used Apriori algorithm with multiple Minsup for rule generation. Since the number of rules that satisfy Minsup and Minconf conditions is high and considering all subset of rules is not possible, we applied the Harmony Search algorithm for finding the best subset of rules that can be used as a classifier. Harmony Search (HS) is a relatively simple yet very efficient evolutionary algorithm. One of the main sections in every population based algorithms is calculating the cost function. For every solution (subset of selected rules) we applied a modified version of the CBA algorithm on training and validation data and assigned the resulted value to the cost function. The statistical and experimental results of applying the proposed method on seventeen benchmark dataset demonstrate that our proposed method outperformed famous algorithms such as tree search, CBA and CPAR in general.

One of the limitations of the proposed method is that it does not gain proper accuracy in datasets with many class number. Another limitation in our study is that we used accuracy measure for comparing the algorithms. Using measures such as precision and recall better reflects the benefits of the proposed method. Our aim in the future is to tackle these problems.

##  Supplemental Information

10.7717/peerj-cs.188/supp-1 Supplemental Information 1 Datasets and Matlab codes

## Additional Information and Declarations

### Data Availability

The following information was supplied regarding data availability: This work uses standard benchmark datasets that can be downloaded from the following addresses: https://sci2s.ugr.es/keel/datasets.php;
https://archive.ics.uci.edu/ml/index.php. The MATLAB code is available as a Supplemental File.
